# Utilitarian Concept of Mind and Mental Health

**DOI:** 10.1192/j.eurpsy.2022.1787

**Published:** 2022-09-01

**Authors:** S. Jain, M. Thirunavukarasu

**Affiliations:** 1 Institute of human behaviour and allied sciences, Psychiatry, Delhi, India; 2 SRM Medical College Hospital And Research Center, Psychiatry, Chennai, India

**Keywords:** Promotion of mental health, mind and mental health, impact on self and impact on others, mental health tool

## Abstract

**Introduction:**

Many classification system and mental health act in many country attempted to define mental illness but mental health perse has not been defined.Some unaddressed question like ” what is diseased in mental illness, what do you treat or set right by treatment, how the psychiatrist say that a pateint is improved and describe or define mental health” are addressed.

**Objectives:**

Working concept for professionals of all allied clinical disciplines. Enable them to understand mental illness and mental health in a uniform and consistent way. Enable all MHP to speak the same language, without room for personal bias. Avoid misconceptions and reduce the stigma with mental illness

**Methods:**

We divided spectrum of mental health into M ***entally Healthy, Not Healthy, Unhealthy and ill*** . Based on two dimension: 1. ***impact on self and 2. Impact on other. Awareness of ones own self, ability to relate well with other and ones own actions are useful to self as well as others*** are the three arms of mental health.

**Results:**

A presentation was given to psychiatrist & allied sciences professional & members from judicairy, technolocrats, industrialist and educationist. Interaction was recorded and analysed, people even gave their responses comments and suggestions by mail and writing.

**Conclusions:**

Mind is defined as a functional concept consists of ***Mood Thought and Intellect*** which is nicely amalgamated in a synchronised manner which always function in unison and the constituents cannot function in isolation. The three arms of the mental health are used to develop a tool to measure the same. All details will be discussed during the presentation.

**Disclosure:**

No significant relationships.

